# WD repeat domain 48 promotes hepatocellular carcinoma progression by stabilizing c‐Myc

**DOI:** 10.1111/jcmm.17583

**Published:** 2022-11-20

**Authors:** Bo Li, Ye‐wei Zhang, Kun Cao, Chao Li, Qian Chen, Yi‐heng Jiang, Lu‐ling Luo, Shi Zuo

**Affiliations:** ^1^ Department of Clinical Medicine Guizhou Medical University Guiyang Guizhou China; ^2^ Department of Hepatobiliary Surgery The Affiliated Hospital of Guizhou Medical University Guiyang Guizhou China; ^3^ Department of General Surgery The First People's Hospital of Fuquan Fuquan Guizhou China

**Keywords:** c‐Myc, deubiquitination, hepatocellular carcinoma, WDR48

## Abstract

The role of protein members containing the WD40 repeat domain in many diseases, including cancer, is well documented. However, the role of WD repeat domain 48 (WDR48) in hepatocellular carcinoma (HCC) and its molecular basis remain to be further investigated. In the present study, we report that WDR48 is downregulated in clinical HCC samples and evaluate the relationship between its expression and clinical features of HCC. In vitro experiments showed that WDR48 positively regulated the proliferation, invasion and metastasis of HCC cells and in vivo experiments showed that downregulation of WDR48 significantly inhibited the tumorigenicity of HCC cells. Mechanistically, WDR48 binds to the proto‐oncogene transcriptional regulator c‐Myc and stabilizes c‐Myc expression by mediating its deubiquitination, thereby enhancing cell proliferation and EMT signalling. Our study demonstrates the oncogenic role of WDR48 and suggests that WDR48 can be an important target in HCC.

## 1 INTRODUCTION

Hepatocellular carcinoma (HCC) accounts for more than 90% of primary liver cancer and is also one of the leading causes of cancer‐related death in the world.^1^ At present, surgical resection, liver transplantation, transcatheter arterial chemoembolization, radiofrequency ablation and other comprehensive treatments are the main treatment options for liver cancer. However, 80% of patients with liver cancer are diagnosed in the middle and late stages due to the concealed onset of liver cancer; invasiveness, metastasis, recurrence and other related characteristics; and the lack of specific early markers.^2^ Although the efficacy of molecular targeted therapy and emerging immunotherapy in treating liver cancer is encouraging, the overall response rate is still poor.^3^ Therefore, actively exploring more effective biomarkers and targets for more accurate diagnosis and more effective treatment is of great significance.

WD Repeat Domain 48 (WDR48), located on human chromosome 3p22.2, encodes a protein of 677 amino acids. It is isolated from the cellular proteins related to proteins interacting with tyrosine kinases in squirrel monkey lymphoid herpesvirus and belongs to the WD40 protein family.^4^ WD40 proteins are usually formed by 4–16 highly conserved WD40 domains, which act as the assembly of various types of molecular machinery. The relationship between their sequence and structure and their relationship with diseases have been widely studied.^5^ As a protein–protein scaffold, the WD40 protein plays an important role in signal transduction, RNA synthesis and processing, cell cycle regulation, apoptosis and so on.^6^ Studies have shown that the WD40 protein is abnormally expressed in many kinds of human malignant tumours, such as liver cancer, colorectal cancer, lung cancer, oesophageal cancer, breast cancer, cervical cancer and so on.^7^, ^8^, ^9^, ^10^ WDR48 has been reported to regulate the activities of ubiquitin‐specific proteases USP1, USP12 and USP46. The WDR48–USP1 complex acts as a regulator during DNA damage, especially in translation synthesis and Fanconi anaemia pathway.^11^, ^12^, ^13^ Recent studies have found that the USP1 complex can downregulate the polyubiquitination of TAK1 and mediate its stability in vitro.^14^ However, few reports exist on the role and mechanism of WDR48 in HCC.

C‐Myc is an important transcriptional regulatory factor, which participates in regulating a variety of genes, thus affecting cell growth, proliferation, apoptosis, metabolism and protein synthesis.^15^, ^16^, ^17^, ^18^ Often maladjusted and highly expressed in most cancers, it is one of the most important oncogenes in HCC. It contributes to hepatocyte proliferation, liver regeneration and tumorigenesis.^19^, ^20^ However, the molecular mechanism of carcinogenesis caused by the functional overactivation of c‐Myc is still unclear and hence needs to be further studied.

In this study, we found that WDR48 was aberrantly upregulated in HCC and positively correlated with poorer survival status, pathological grade and prognosis. WDR48 promoted the proliferation of HCC cells in vitro and in vivo. In terms of the mechanism, WDR48 inhibited the ubiquitin degradation of c‐Myc by binding to it, thus promoting the occurrence and development of HCC. Therefore, this study discussed the effect of WDR48 on the occurrence and development of HCC and its potential mechanism and provided a theoretical basis for WDR48 to become a useful target for treating HCC.

## 2 MATERIALS AND METHODS

### 2.1 Clinical specimens

Twenty‐eight patients with HCC from the Affiliated Hospital of Guizhou Medical University, who underwent HCC radical resection, were enrolled in the study after obtaining informed consent. The diagnosis of HCC was confirmed by a histopathologist in each patient sample. At the same time, the scheme used in this study was approved by the Ethics Review Committee of our hospital.

### 2.2 Cell culture

Huh7, HCCLM3, Hep3B and HepG2 cells were purchased from the Cell Bank of the Chinese Academy of Sciences. LO2 cells were from the Cancer Institute of Southern Medical University. All the cell lines were cultured in DMEM supplemented with 10% FBS in a 37C and 5% CO2 incubator.

### 2.3 Plasmid construction and cell transfection

Aiming at WDR48 knockdown and overexpression, siRNA (Table S2) and plasmids were obtained from RiboBio Corporation (Guangzhou, China) and from Vigene Biosciences Corporation (Shandong, China), respectively. siRNA and plasmid were transfected with Lipofectamine 2000 and Lipofectamine 3000, respectively. The cells were harvested 24–48 h after transfection for further experiment.

### 2.4 Q‐PCR and RT‐PCR

Total RNA was extracted using a Cell Total RNA Isolation Kit (Foregene) and RNA was transcribed into cDNA using a reverse transcription kit (TaKaRa). The cDNA templates were then used for amplification using specific primers (Table S3). A Bio‐Rad CFX 96 detection system was used for qPCR. An SYBR Premix Ex Taq II kit (TaKaRa) was used for RT‐PCR on a Bio‐Rad T100 detection system. β‐actin was used as the sample control. The 2^−ΔΔCt^ method was used to evaluate the relative abundance of genes.

### 2.5 Western blot analysis

The total protein was extracted from tumour samples and cells using the lysis buffer, and the protein was quantified. The same amounts of protein samples were separated by sodium dodecyl sulphate‐polyacrylamide gel electrophoresis and transferred to a polyvinylidene fluoride (PVDF) membrane, which was co‐incubated with a specific primary antibody (4C, overnight). The primary antibodies including anti‐WDR48, c‐Myc, CCND1, N‐cadherin, E‐cadherin, Vimentin and GAPDH are listed in Table S4. Proteins were detected using a ChemiDocXRS+ molecular imager (Bio‐Rad).

### 2.6 Follow‐up analysis of cycloheximide

Cycloheximide (CHX, Selleck) was resuspended in DMSO (200 mM) and stored at –20C. After transfection, the cells were incubated with 50 μg/ml CHX at 37C in 5% CO_2_ for different time gradients, and the system volume was 2 ml. Subsequently, they were collected and further analysed by Western blot analysis.

### 2.7 Co‐immunoprecipitation

A Pierce Co‐Immunoprecipitation (Co‐IP) kit (ThermoScientific) was used for Co‐IP following the manufacturer's protocols. In short, the total protein was extracted from the cell, and its concentration was determined. A total of 2 mg proteins were incubated overnight with 5 μg specific antibody or IgG at 4C. After elution, the recovered proteins were analysed by Western blot analysis or Coomassie brilliant blue staining. Anti‐IgG was used as a negative control.

### 2.8 Analysis of migration, invasion and wound healing

Transwell assay was used to determine cell migration and invasion. The cells (1 × 105) were inoculated into 100 μl of serum‐free DMEM with or without Matrigel (BD Biosciences Pharmingen) in the upper chamber of Transwell; the lower chamber was filled with DMEM containing 10% FBS. The Transwell chamber was incubated in a humidified incubator at 37C and in the presence of 5% CO_2_ for 24 h. The cells in the upper chamber of Transwell were removed. The bottom of Transwell was fixed with 4% paraformaldehyde for 20 min and stained with 0.1% crystal violet for 10 min. Three visual fields were randomly selected from each membrane to calculate the average number of invading cells in each sample. As for wound healing, the cell plank was grown into a fused monolayer in a six‐hole plate, and the tip of the 1‐ml pipette was slid across the cell monolayer to form a linear wound. The process of cell migration was observed under the microscope after 0 and 48 h.

### 2.9 Determination of cell viability

Huh7 and LM3 cells were inoculated into 96‐well plates at a density of 3 × 103 cells per well. The cell viability was evaluated using a cell counting kit 8 (CCK‐8; Dojindo) kit. After 0, 24, 48 and 72 h of growth, 10 μl of CCK‐8 was added to each well and incubated for 2 h. The absorbance was measured at 450 nm using an enzyme labelling instrument (Thermo Fisher Multiskan Sky). Each sample was analysed five times. The experiment was repeated at least three times.

### 2.10 EdU incorporation analysis

An Apollo567 in vitro imaging kit was purchased from RiboBio Corporation for EdU incorporation analysis. After culturing with EdU (10 μM) for 2 h, the cells were fixed with paraformaldehyde (4%), permeated with Triton X‐100 (0.2%) and co‐stained with 4‐diamidino‐2‐phenylindole (DAPI, 5 μg/ml) and Apollo fluorescent dye.

### 2.11 Immunofluorescence staining

The cells were washed three times with PBS, fixed with 4% paraformaldehyde for 15 min and permeated with 0.3% TritonX‐100 for 30 min. After three washes with PBS, the samples were blocked with normal goat serum for 1 h at room temperature and incubated with different antibodies in normal goat serum overnight at 4C. Then, at room temperature, the cells were incubated with the secondary antibody for 1 h and incubated with DAPI for 10 min. The cells were washed with PBS three times and photographed with a confocal microscope (Zeiss). Primary antibodies included anti‐Flag and c‐Myc. The antibodies are listed in Table S4.

### 2.12 Cycloheximide (CHX) chase assay

Cycloheximide (Selleck) was suspended in DMSO (200 mM) before the experiment and stored at −20°C. After the same amount of cells were plated and transferred forward, the concentration was 50 μg/ml and the system was 2 ml of CHX, and then, the cells were incubated at different time gradients. Then, protein from the cell extract were collected for Western blotting analysis.

### 2.13 Animal experiment

The animal experiment accorded with the requirements of the Animal Research Committee of the Academic Medical Center of Southern Medical University and the International Guidelines for Animal Care and Maintenance. The subcutaneous xenotransplantation mouse model was used to evaluate tumour growth. HCCLM3 cells were subcutaneously injected into the right side of 4‐week‐old BALB/c male nude mice. When the diameter of the tumour reached 3 mm, the mice were randomly divided into two groups: experimental group (si‐WDR48) and control group (si‐NC). In vivo purified siRNA (RiboBio) modified using 2'Ome + 5'Chol was used for siRNA transfection. Buffered with 50 μl of normal saline, each tumour was locally injected with 5 nmol siRNA twice a week for 3 weeks. After 3 weeks, the tumour was harvested, weighed and processed for further experiment.

### 2.14 Statistical analysis

SPSS 25.0 (SPSS) was used for statistical analysis. The data were expressed as mean ± standard deviation (SD) from at least three independent experiments. The Student's *t*‐test or Tukey's multiple comparison test was used for comparison between the two groups, and single‐factor analysis of variance was used for multi‐group comparison. Survival analysis was performed using the Kaplan–Meier method and logarithmic rank test. Cox's proportional hazard regression model was used to analyse independent prognostic factors. *p* < 0.05 indicated a statistical significance (*p < 0.05, **p < 0.01, ***p < 0.001).

## 3 RESULTS

### 3.1 WDR48 was aberrantly upregulated in HCC and associated with poor prognosis

**FIGURE 1:**
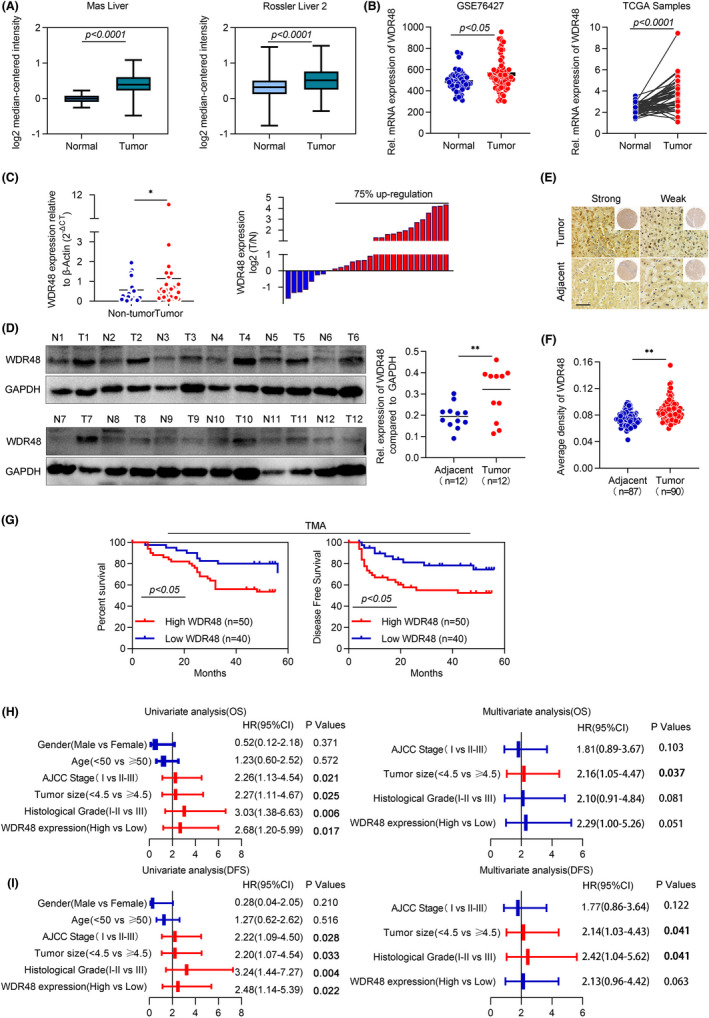
Expression of WDR48 was upregulated and associated with poor prognosis. (A and B) mRNA expression level of WDR48 in HCC was analysed using Oncomine, GEO and TCGA databases. (C) The transcription level of WDR48 was detected by RT‐qPCR in 28 pairs of HCC tissues and adjacent non‐tumour tissues. The results showed that 21 (75%) of them were significantly upregulated. (D) The expression level of WDR48 protein was detected by Western blot in 12 pairs of HCC tissues and adjacent non‐tumour tissues. (E and F) Representative images of tissue microarray stained with anti‐WDR48 by IHC, and the expression of WDR48 in tumour and adjacent samples was detected using an average optical density score. Scale, 100 μm. (G) Kaplan–Meier survival analysis of Overall survival and Disease‐free survival of 90 patients with HCC based on the WDR48 score data. (H and I) Univariate and multivariate Cox regression analyses of different clinicopathological features of patients with HCC.

We found that the transcription level of WDR48 in HCC was upregulated compared with normal samples in Oncomine, GEO and TCGA databases (Figure 1A,B). We detected the expression level of WDR48 (Figure 1C) in 28 pairs of human HCC samples by q‐PCR to further determine the expression of WDR48 in HCC. We found that WDR48 expression was upregulated in 75% tumour tissues compared with their adjacent non‐tumour tissues. Similar results (Figure 1D) were observed by Western blot analysis in 12 fresh HCC specimens and adjacent tissues. Next, we performed immunohistochemical staining for the expression of WDR48 through a tissue microarray containing 90 pairs of HCC samples. As shown in the figure, the average optical density of WDR48 protein in tumour tissues was stronger than that in adjacent tissues (Figure 1E,F). The Kaplan–Meier analysis showed that patients with higher WDR48 expression had shorter Overall survival and Disease‐free survival time (Figure 1G) compared with patients with low expression. At the same time, the correlation between the level of WDR48 and the characteristics of clinical cases was further analysed; it was found that the expression level of WDR48 was significantly correlated with the survival status and pathological grade (Table S1). Importantly, the univariate analysis showed that WDR48 expression was an independent indicator of Overall survival and Disease‐free survival (Figure 1H,I) in patients with HCC. These results indicated that the high expression of WDR48 was closely associated with tumour progression.

### 3.2 WDR48 promoted the proliferation, invasion and metastasis of HCC cells

**FIGURE 2:**
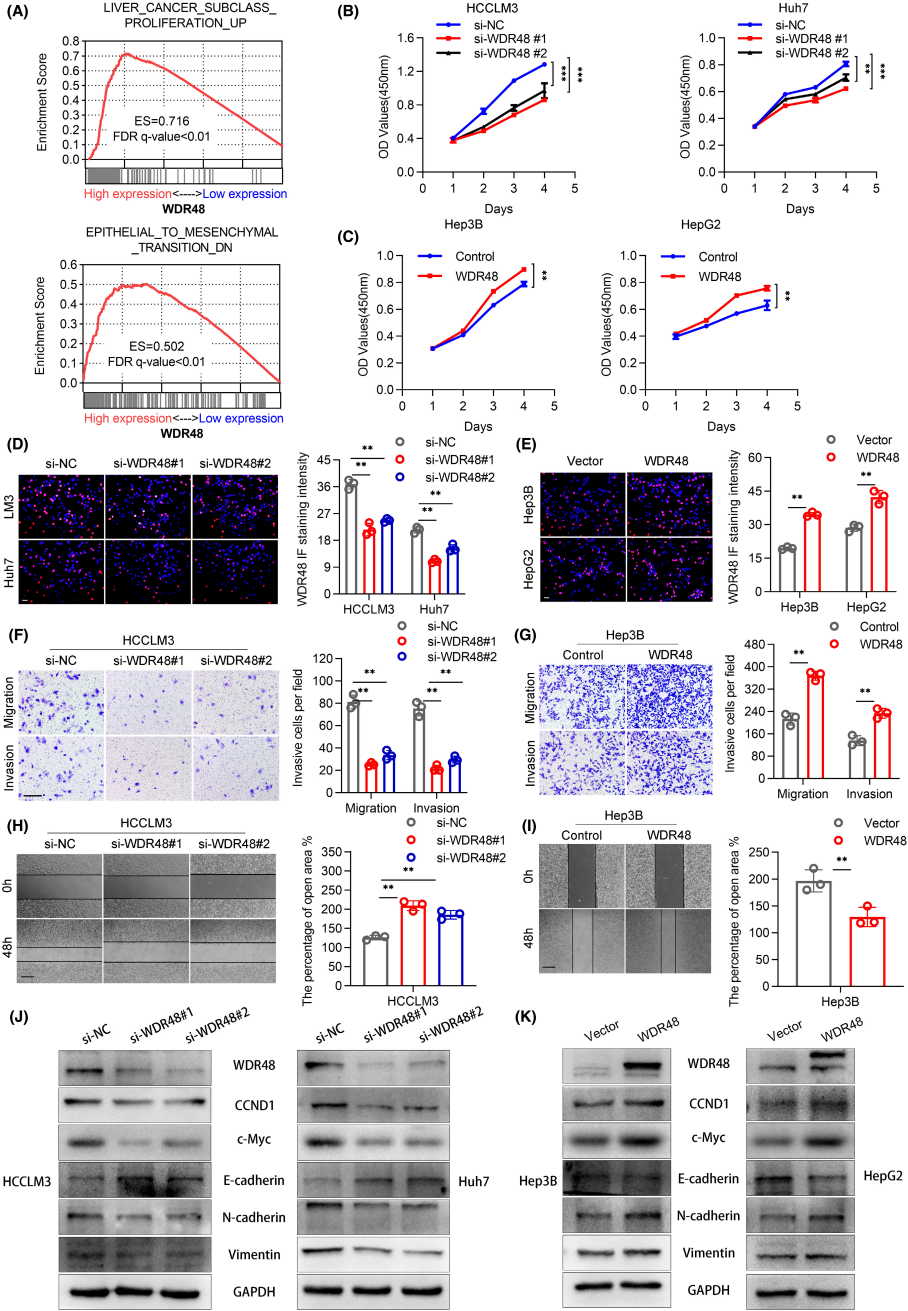
Under or overexpression of WDR48 affected the proliferation, metastasis and invasion of HCC. (A) Predefined genomes involved in cell proliferation and metastasis were significantly enriched in HCC with a high level of WDR48, as detected by GSEA enrichment analysis. (B and C) WDR48 knockdown inhibited the cell viability of HCCLM3 and Huh7 cells, while the overexpression of WDR48 promoted the cell viability of Hep3B and HepG2, as detected using CCK‐8. (D and E) EdU analysis of HCCLM3 and Huh7 cells with low WDR48 expression and Hep3B and HepG2 cells with overexpression of WDR48. Scale, 200 μm. (F and G) Migration and invasion of hepatoma cells treated with si‐WDR48 and WDR48 plasmids and corresponding controls were evaluated by Transwell assay, Boyden assay and (H and I) wound‐healing assay. Scale, 200 μm. (J and K) The expression levels of CCND1, c‐Myc, N‐cadherin, E‐cadherin and Vimentin in HCCLM3, Huh7, Hep3B and HepG2 cells were detected by Western blot. Student's *t*‐test. Mean ± SD (*p < 0.05; **p < 0.01).

We performed GSEA enrichment analysis to explore the biological role of WDR48 in the development of HCC, revealing that predefined genomes involved in cell proliferation and metastasis were significantly enriched (Figure 2A) in HCC with a high level of WDR48. siRNA was used to inhibit the expression of WDR48 in HCCLM3 and HUH7 cells, and the WDR48 plasmid was introduced into Hep3B and HepG2 cells (Figure S1A–D). CCK‐8 and EdU infiltration experiments showed that the knockdown of WDR48 significantly inhibited the viability of HCC cells and slowed down cell proliferation, while the overexpression of WDR48 had the opposite effect (Figure 2B–E). Transwell device and Borden chamber coated with the matrix glue were used to further understand the effect of WDR48 on the migration and invasion of HCC cells. WDR48 knockdown reduced the migration and invasion of HCC cells. The overexpression of WDR48 promoted its migration and invasion (Figure 2F,G, Figure S2A,B). At the same time, WDR48 knockdown significantly inhibited wound healing in the wound‐healing test, while WDR48 overexpression had the opposite effect. (Figure 2H,I, Figure S2C,D). In addition, we found that the knockdown of WDR48 decreased the protein level of the important proto‐oncogenic transcription factor c‐Myc and its downstream target gene CCND1, decreased the level of mesenchymal markers N‐cadherin and Vimentin and increased the level of epithelial marker E‐cadherin. The overexpression of WDR48 also had the opposite effect (Figure 2J,K). These results suggested that WDR48 promoted the proliferation, migration and invasion of HCC.

### 3.3 WDR48 interacts with c‐Myc

**FIGURE 3:**
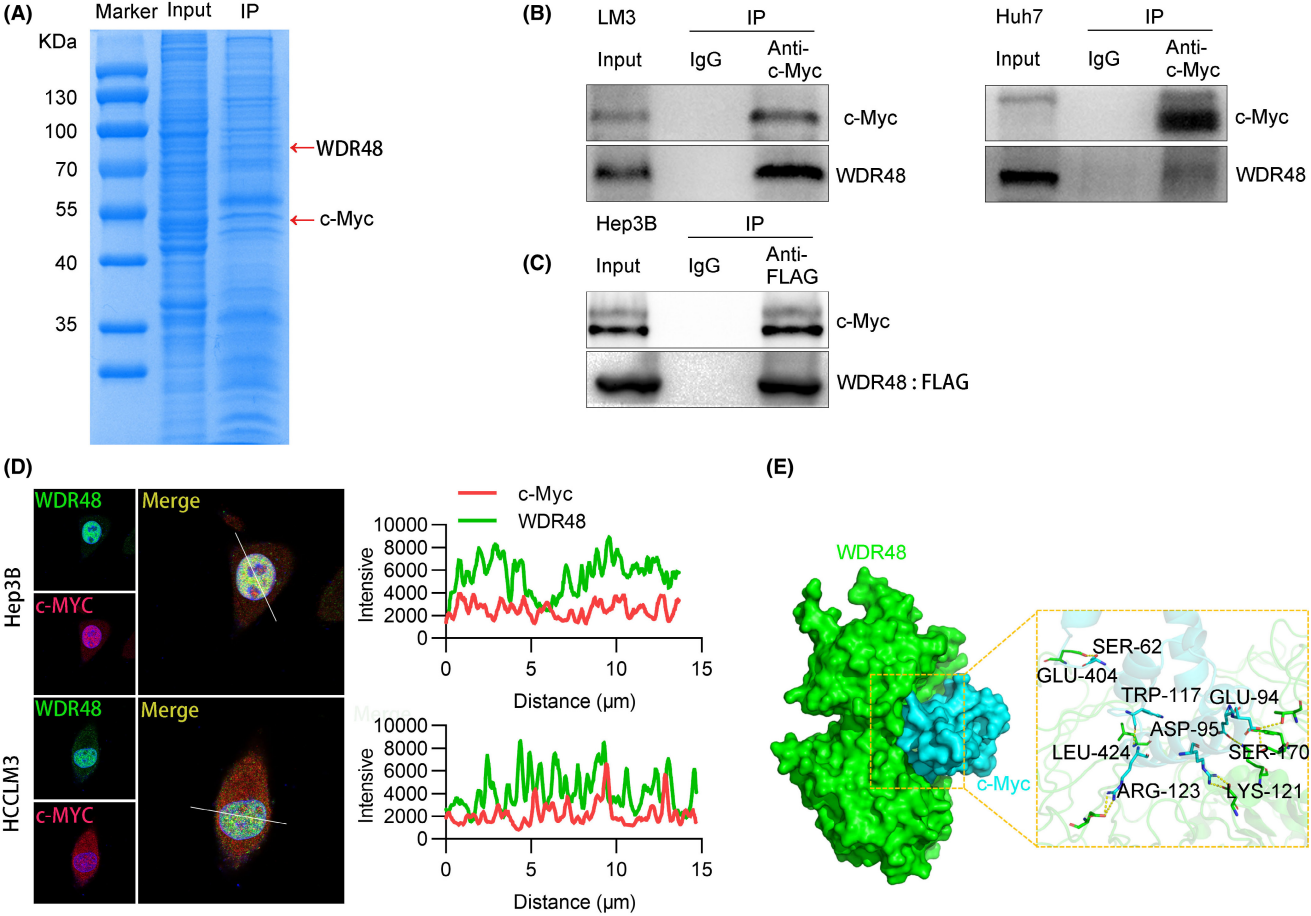
WDR48 and c‐Myc interaction. (A) Coomassie brilliant blue staining showed the proteins interacting with WDR48 and the molecular weights of WDR48 and c‐Myc in Hep3B cells. (B) Interaction between endogenous WDR48 and c‐Myc was detected by Co‐IP. (C) Interaction between exogenous WDR48 and c‐Myc was detected by Co‐IP. (D) Co‐localization of WDR48 and c‐Myc in Hep3B and HCCLM3 was evaluated by immunofluorescence staining. (E) Molecular docking of WDR48 and c‐Myc.

The key role of WDR48 in tumour progression urged us to determine the mechanism by which WDR48 promoted cell growth and metastasis. We further studied the molecular mechanism of WDR48‐mediated progression of HCC through the prediction of BioGrid and HitPredict data sets and found that c‐Myc might interact with the WDR48/USP1 complex. Many studies showed that c‐Myc played an important role in the progression of HCC. Therefore, we studied whether WDR48 interacted with c‐Myc; exogenous and endogenous Co‐IP proved the interaction between WDR48 and c‐Myc (Figure 3A–C). Immunofluorescence showed that WDR48 and c‐Myc proteins were mainly co‐located in the nucleus (Figure 3D). The protein docking model also showed the interaction between the two proteins (Figure 3E). Overall, these data suggested that WDR48 played a role in HCC through its interaction with c‐Myc.

### 3.4 WDR48 stabilized c‐Myc by mediating its deubiquitination

**FIGURE 4:**
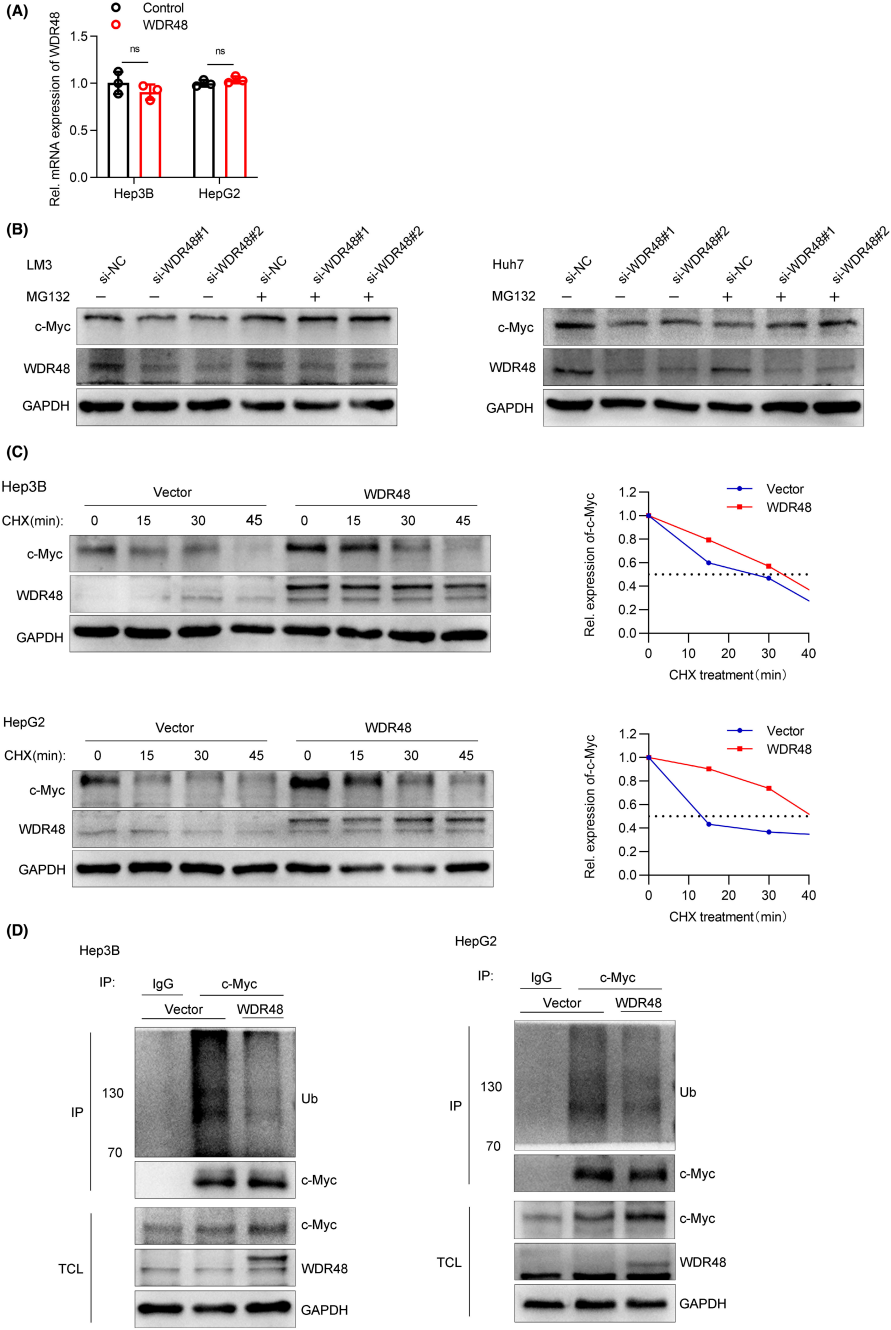
WDR48 inhibited the degradation of c‐Myc by mediating its deubiquitination. (A) The mRNA level of c‐Myc was detected by RT‐qPCR after WDR48 overexpression. (B) Cell lysates were prepared from HCCLM3 and Huh7 cells treated with or without MG132 for 8 h and then immunoblotted with anti‐WDR48 and c‐Myc. (C) Hep3B and HepG2 cells were treated with CHX for a specified time. The cell lysates were immunoblotted with designated antibodies. (D) Hep3B or HepG2 cells were transfected with WDR48 plasmid or control plasmid for 48 h and treated with the proteasome inhibitor MG132 for 8 h. The cell lysates were immunoprecipitated with anti‐c‐Myc antibodies and then immunoblotted with anti‐ubiquitin antibodies, while total cell lysates were immunoblotted with antibodies of WDR48, c‐Myc and GAPDH. Mean ± SD (ns *p* > 0.05).

Because of the interaction between WDR48 and c‐Myc, we tried to figure out how WDR48 was involved in regulating the expression of c‐Myc. Interestingly, we found that the protein level of c‐Myc was downregulated after WDR48 knockdown in Western blot detection, and the protein level of c‐Myc was upregulated while the mRNA level of c‐Myc was not affected after WDR48 overexpression (Figure 4A). Therefore, we suspected that it was not possible for WDR48 to regulate the transcriptional level of c‐Myc. To test whether WDR48 regulated c‐Myc at the post‐transcriptional level, we found that proteasome inhibitor MG132 prevented the decrease in the c‐Myc protein level (Figure 4B) caused by WDR48 consumption. Therefore, we suspected that WDR48 stabilized c‐Myc by inhibiting the degradation of c‐Myc by the proteasome. To confirm the aforementioned result, a CHX tracing analysis was carried out to detect the half‐life of c‐Myc treated with WDR48 overexpression. We found the shortened half‐life of c‐Myc proteins and a remarkable accumulation of c‐Myc proteins in WDR48‐overexpressing cells with CHX treatment (Figure 4C). WDR48 often binds to a variety of deubiquitinating enzymes and regulates its deubiquitination activity, and hence we speculated that WDR48 might affect the stability of c‐Myc by participating in the deubiquitination of c‐Myc. C‐Myc was immunoprecipitated with a specific anti‐c‐Myc antibody, and its ubiquitin status (Figure 4D) was analysed using an anti‐ubiquitin antibody. As expected, the overexpression of WDR48 significantly reduced the ubiquitin level of c‐Myc. These results indicated that WDR48 regulated protein stability by affecting the ubiquitination level of c‐Myc.

### 3.5 C‐Myc participated in WDR48 cells to promote the proliferation, invasion and metastasis of HCC

**FIGURE 5:**
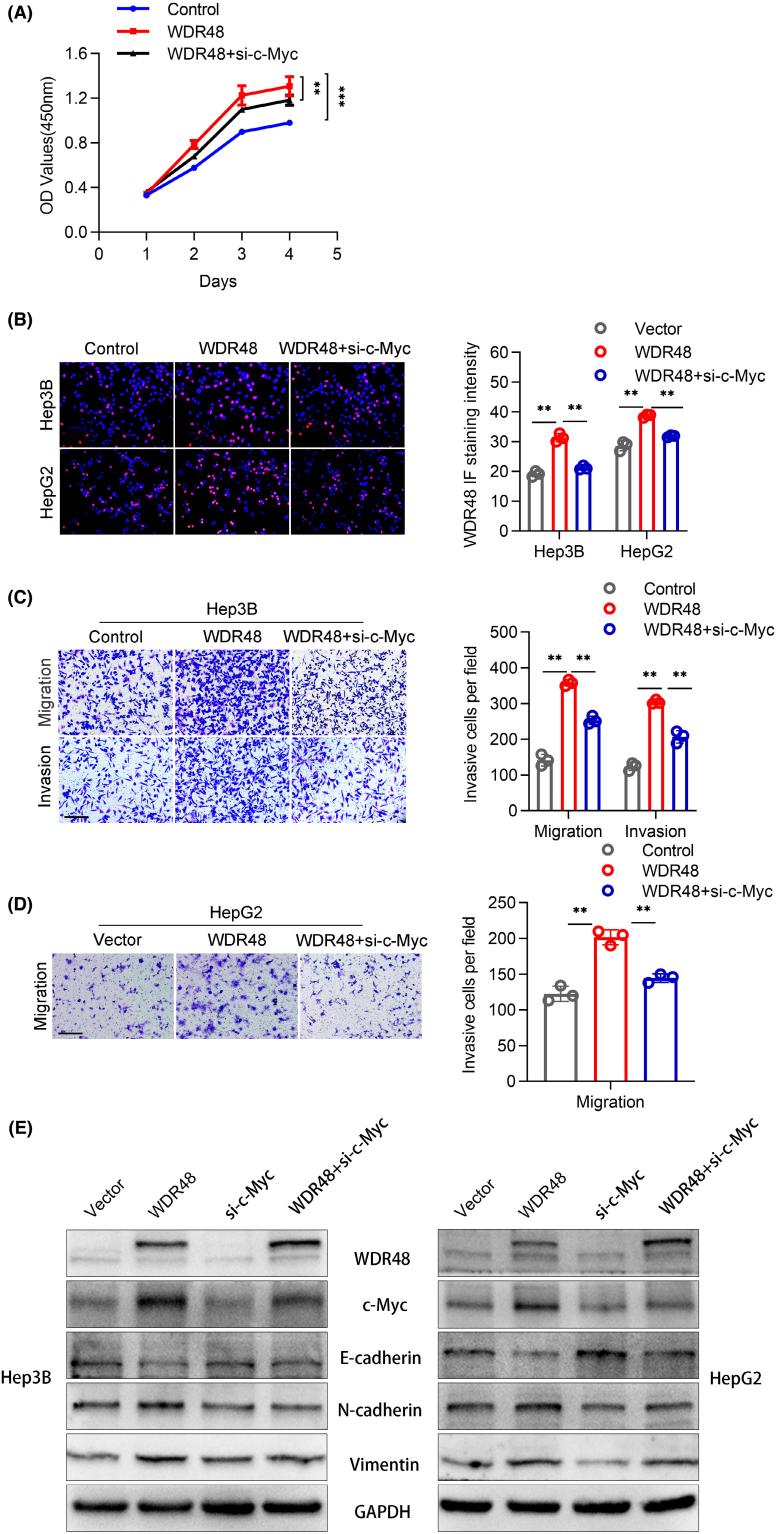
Knockdown of c‐Myc saved the proliferation, invasion and metastasis of HCC caused by the overexpression of WDR48. (A‐D) MTT, Transwell and Boyden assays were used to evaluate cell viability and metastasis in Vector, WDR48‐OE and WDR48‐OE + si‐c‐Myc groups. (E) Western blot analysis was used to evaluate the levels of WDR48, c‐Myc, E‐cadherin, N‐cadherin and Vimentin in Hep3B or HepG2 cells treated with vector, WDR48‐OE, si‐c‐Myc and WDR48‐OE + si‐c‐Myc. Student's *t*‐test. Mean ± SD (*p < 0.05; **p < 0.01).

We further examined whether c‐Myc was involved in the role of WDR48 in HCC. We found that the transient transfection of si‐c‐Myc into WDR48‐overexpressing cells decreased CCK‐8‐based cell viability and EdU‐based cell proliferation (Figure 5A,B). In addition, Transwell and Boyden assays showed that c‐Myc knockdown also reduced the migration and invasion abilities of WDR48‐overexpressing cells (Figure 5C,D). At the same time, si‐c‐Myc eliminated the promotion of N‐cadherin and Vimentin mediated by WDR48 and the inhibition of E‐cadherin (Figure 5E), indicating that c‐Myc was involved in the process of WDR48 regulating the progression of HCC. In general, our data showed that WDR48 could be used as an important deubiquitination complex cofactor, to some extent, by binding to c‐Myc and mediating its deubiquitinated degradation process, thus playing an important role in the progression of HCC.

### 3.6 WDR48 promoted the growth of HCC in vivo

**FIGURE 6:**
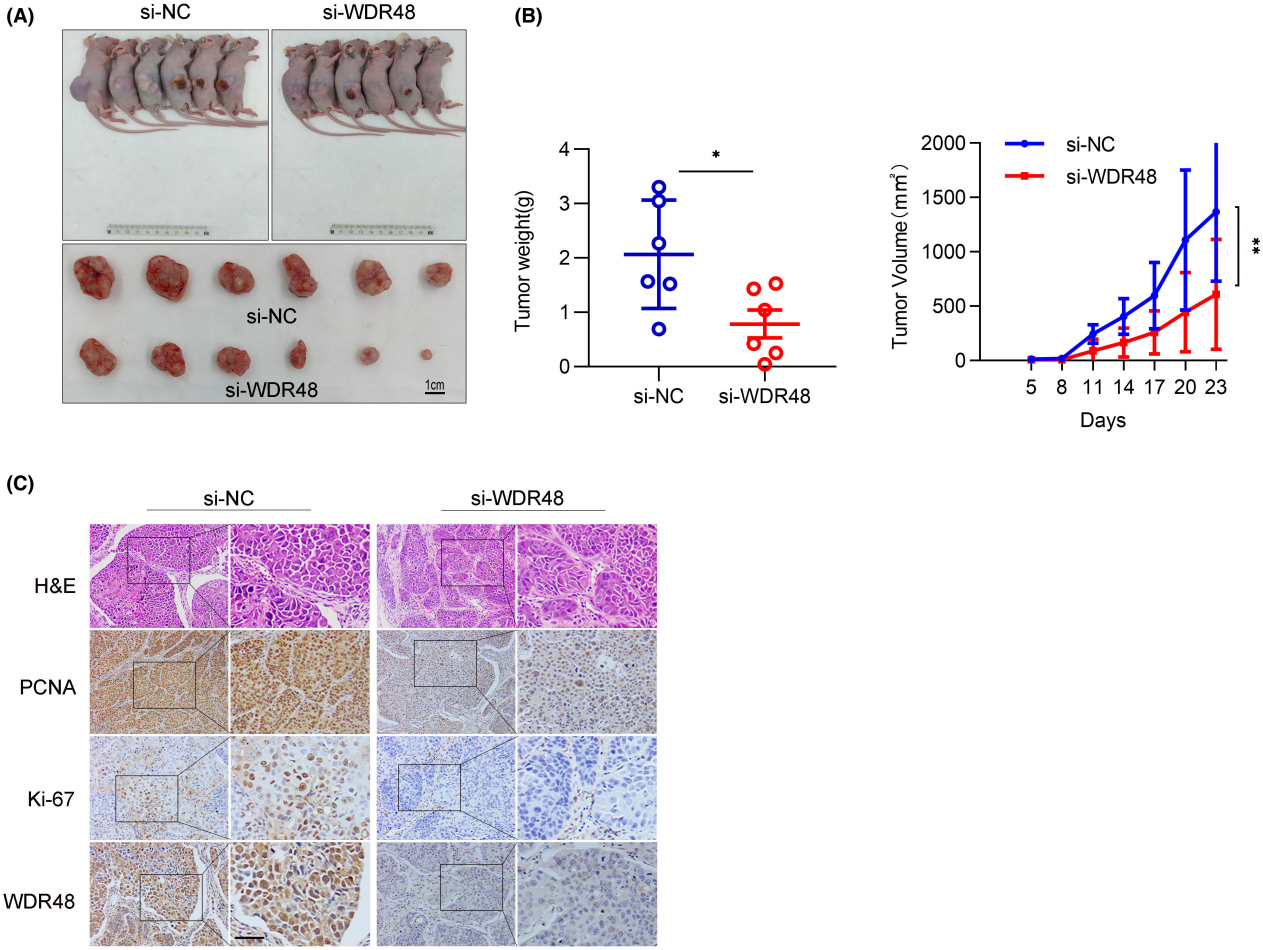
WDR48 promoted the growth of HCC in vivo. (A) In vivo effect of si‐WDR48 was evaluated by injecting control or si‐WDR48 once every 3 days in a xenograft mouse model with tumours derived from HCCLM3 cells. (B) Growth curve and weight analysis of si‐NC or si‐WDR48 HCCLM3 xenografts in nude mice. (C) A representative image of immunohistochemical staining of xenografts and expression of Ki67, PCNA and WDR48 was shown. Scale, 100 μm.

HCCLM3 was subcutaneously transplanted into nude mice to establish a xenograft model so as to determine the role of WDR48 in the malignant progression of HCC in vivo. When the subcutaneous tumour of nude mice grew to about 3 mm in diameter 5 days after inoculation, si‐NC or si‐WDR48 was injected into the developing tumour (Figure 6A). The results showed a significant decrease in tumour growth and weight (Figure 6B) in the group injected with si‐WDR48. IHC showed the expression of WDR48 was downregulated and the expression of Ki67 and PCNA decreased in the si‐WDR48 group compared with the control group (Figure 6C). These results suggested that WDR48 promoted the growth of HCC.

## 4 DISCUSSION

WDR48 is a member of the WD40 protein family, which consists of an N‐terminal domain consisting of eight WD repeats and a hypothetical C‐terminal crimp domain. Many studies reported that the WD40 protein family was associated with cancer. However, few reports exist on the role of WDR48 in HCC. Based on Oncomine, GEO and TCGA databases, we found that WDR48 was upregulated in HCC. In the present study, the expression of WDR48 increased in patients with HCC and was significantly correlated with the survival status and pathological grade. In addition, patients with increased expression of WDR48 had a poorer prognosis. Subsequently, we found that the knockdown of WDR48 inhibited the proliferation, invasion and metastasis of HCC cells in vivo and in vitro, while the overexpression of WDR48 had the opposite result. Therefore, we proved that WDR48 played an important role in the development of HCC.

C‐Myc is an important proto‐cancer transcription factor, which plays an important role in HCC cell proliferation and tumorigenesis.^21^ The inactivation of c‐Myc can induce the regression of invasive HCC.^22^ In our study, we found that WDR48 affected the occurrence and development of HCC by regulating the activation of c‐Myc. After the use of si‐c‐Myc in hepatoma cells, the proliferation and EMT signal of hepatoma cells mediated by the overexpression of WDR48 decreased.

We further investigated how WDR48 affected the activation of c‐Myc. Based on the prediction of BioGrid and HitPredict data sets, we found that c‐Myc might interact with WDR48 high‐binding ubiquitin‐specific peptidase 1 (USP1). WDR48 was also known as a USP1‐related factor 1.^7^ The interaction of the WDR48/USP1 complex with the substrate mainly depended on the C‐terminal domain of the WDR48‐targeting substrate, and WDR48 was distributed in the cytoplasm and nucleus. We detected the correlation between WDR48 and c‐Myc by immunofluorescence and Co‐IP. As expected, we found that WDR48 had no significant effect on the mRNA level of c‐Myc. In vivo, WDR48 interacted with c‐Myc and participated in the inhibition of c‐Myc ubiquitin and degradation. We speculated that the formation of the complex of WDR48 and USP1 affected the ubiquitin degradation of c‐Myc by increasing its deubiquitination activity, which was worthy of further exploration. However, the regulation of protein stability of c‐Myc through the ubiquitin–proteasome system is also one of the mechanisms to control its function.^23^ Therefore, the deubiquitination effect of WDR48 on c‐Myc is very important for WDR48‐mediated hepatoma proliferation and EMT.

In short, our study confirmed that WDR48 played the role of proto‐oncogene in HCC. WDR48 mediated its ubiquitination and degradation by binding to c‐Myc, thus promoting the proliferation of HCC. Our study provided a new insight for understanding the pathogenesis of HCC.

## AUTHOR CONTRIBUTIONS

**Bo Li:** Data curation (equal); investigation (equal); methodology (equal); software (equal); validation (equal); writing – original draft (equal). **Shi Zuo:** Funding acquisition (equal); resources (equal). **Ye‐wei Zhang:** Project administration (equal). **Kun Cao:** Methodology (equal). **Chao Li:** Resources (equal). **Qian Chen:** Investigation (equal). **Yi‐hemg Jiang:** Supervision (equal). **Lu‐ling Luo:** Software (equal).

## FUNDING INFORMATION

This work was financed by grants received from the 12th Special Fund for Young Scientist of Guizhou Province [(2019)5628], the National Natural Science Foundation of China (NSFC) Cultivation Program of Affiliated Hospital of Guizhou Medical University (gyfynsfc‐2021‐34).

## CONFLICT OF INTEREST

The authors declare that the research was conducted in the absence of any commercial or financial relationships that could be construed as a potential conflict of interest.

## Supporting information


Figure S1

Figure S2

Table S1‐S4

## Data Availability

The data sets presented in this study can be found in online repositories. The names of the repository/repositories and accession number(s) can be found in the article/Supplementary Material.

## References

[1] Bray F , Ferlay J , Soerjomataram I , Siegel RL , Torre LA , Jemal A . Global cancer statistics 2018: GLOBOCAN estimates of incidence and mortality worldwide for 36 cancers in 185 countries. CA Cancer J Clin. 2018;68(6):394‐424. doi:10.3322/caac.21492 30207593

[2] Forner A , Reig M , Bruix J . Hepatocellular carcinoma. Lancet. 2018;391(10127):1301‐1314. doi:10.1016/S0140-6736(18)30010-2 29307467

[3] Kuczynski EA , Lee CR , Man S , Chen E , Kerbel RS . Effects of sorafenib dose on acquired reversible resistance and toxicity in hepatocellular carcinoma. Cancer Res. 2015;75(12):2510‐2519. doi:10.1158/0008-5472.CAN-14-3687 25908587 PMC6485661

[4] Olazabal‐Herrero A , Peters GJ , Giovannetti E , Rodriguez JA . WDR48 (WD repeat domain 48). Atlas Genet Cytogenet Oncol Haematol. 2018;22(2):44‐50. doi:10.4267/2042/68759

[5] Jain BP , Pandey S . WD40 repeat proteins: signalling scaffold with diverse functions. Protein J. 2018;37(5):391‐406. doi:10.1007/s10930-018-9785-7 30069656

[6] Song R , Wang ZD , Schapira M . Disease association and Druggability of WD40 repeat proteins. J Proteome Res. 2017;16(10):3766‐3773. doi:10.1021/acs.jproteome.7b00451 28956604

[7] Zhao Y , Xue C , Xie Z , Ouyang X , Li L . Comprehensive analysis of ubiquitin‐specific protease 1 reveals its importance in hepatocellular carcinoma. Cell Prolif. 2020;53(10):e12908. doi:10.1111/cpr.12908 32951278 PMC7574869

[8] Yuan Y , Qi G , Shen H , et al. Clinical significance and biological function of WD repeat domain 54 as an oncogene in colorectal cancer. Int J Cancer. 2019;144(7):1584‐1595. doi:10.1002/ijc.31736 29987896

[9] Li Y , Chen F , Shen W , et al. WDR74 induces nuclear β‐catenin accumulation and activates Wnt‐responsive genes to promote lung cancer growth and metastasis. Cancer Lett. 2020;471:103‐115. doi:10.1016/j.canlet.2019.12.011 31838084

[10] Li Y , Zhou Y , Li B , et al. WDR74 modulates melanoma tumorigenesis and metastasis through the RPL5‐MDM2‐p53 pathway. Oncogene. 2020;39(13):2741‐2755. doi:10.1038/s41388-020-1179-6 32005977

[11] Cohn MA , Kowal P , Yang K , et al. A UAF1‐containing multisubunit protein complex regulates the Fanconi anemia pathway. Mol Cell. 2007;28(5):786‐797. doi:10.1016/j.molcel.2007.09.031 18082604

[12] Gangula NR , Maddika S . WD repeat protein WDR48 in complex with deubiquitinase USP12 suppresses Akt‐dependent cell survival signaling by stabilizing PH domain leucine‐rich repeat protein phosphatase 1 (PHLPP1). J Biol Chem. 2013;288(48):34545‐34554. doi:10.1074/jbc.M113.503383 24145035 PMC3843068

[13] Hodul M , Ganji R , Dahlberg CL , Raman M , Juo P . The WD40‐repeat protein WDR‐48 promotes the stability of the deubiquitinating enzyme USP‐46 by inhibiting its ubiquitination and degradation. J Biol Chem. 2020;295(33):11776‐11788. doi:10.1074/jbc.RA120.014590 32587090 PMC7450142

[14] Han D , Wang L , Chen B , et al. USP1‐WDR48 deubiquitinase complex enhances TGF‐β induced epithelial‐mesenchymal transition of TNBC cells via stabilizing TAK1. Cell Cycle. 2021;20(3):320‐331. doi:10.1080/15384101.2021.1874695 33461373 PMC7889205

[15] Grandori C , Cowley SM , James LP , Eisenman RN . The Myc/max/mad network and the transcriptional control of cell behavior. Annu Rev Cell Dev Biol. 2000;16:653‐699. doi:10.1146/annurev.cellbio.16.1.653 11031250

[16] Dang CV . MYC on the path to cancer. Cell. 2012;149(1):22‐35. doi:10.1016/j.cell.2012.03.003 22464321 PMC3345192

[17] Wang Y , Chen S , Jiang Q , et al. TFAP2C facilitates somatic cell reprogramming by inhibiting c‐Myc‐dependent apoptosis and promoting mesenchymal‐to‐epithelial transition. Cell Death Dis. 2020;11(6):482. doi:10.1038/s41419-020-2684-9 32587258 PMC7316975

[18] Liu JY , Chen YJ , Feng HH , et al. LncRNA SNHG17 interacts with LRPPRC to stabilize c‐Myc protein and promote G1/S transition and cell proliferation. Cell Death Dis. 2021;12(11):970. doi:10.1038/s41419-021-04238-x 34671012 PMC8528917

[19] Qu A , Jiang C , Cai Y , et al. Role of Myc in hepatocellular proliferation and hepatocarcinogenesis. J Hepatol. 2014;60(2):331‐338. doi:10.1016/j.jhep.2013.09.024 24096051 PMC3909877

[20] Murakami H , Sanderson ND , Nagy P , Marino PA , Merlino G , Thorgeirsson SS . Transgenic mouse model for synergistic effects of nuclear oncogenes and growth factors in tumorigenesis: interaction of c‐myc and transforming growth factor alpha in hepatic oncogenesis. Cancer Res. 1993;53(8):1719‐1723. 8467484

[21] Kaposi‐Novak P , Libbrecht L , Woo HG , et al. Central role of c‐Myc during malignant conversion in human hepatocarcinogenesis. Cancer Res. 2009;69(7):2775‐2782. doi:10.1158/0008-5472.CAN-08-3357 19276364 PMC2680077

[22] Shachaf CM , Kopelman AM , Arvanitis C , et al. MYC inactivation uncovers pluripotent differentiation and tumour dormancy in hepatocellular cancer. Nature. 2004;431(7012):1112‐1117. doi:10.1038/nature03043 15475948

[23] Salghetti SE , Kim SY , Tansey WP . Destruction of Myc by ubiquitin‐mediated proteolysis: cancer‐associated and transforming mutations stabilize Myc. EMBO J. 1999;18(3):717‐726. doi:10.1093/emboj/18.3.717 9927431 PMC1171164

