# Supplementary figures and images for: WD repeat domain 48 promotes hepatocellular carcinoma progression by stabilizing c‐Myc
Source: J Cell Mol Med. 2022 Nov 20;26(23):5755–66. doi: 10.1111/jcmm.17583 (PMC9716212; doi:10.1111/jcmm.17583)

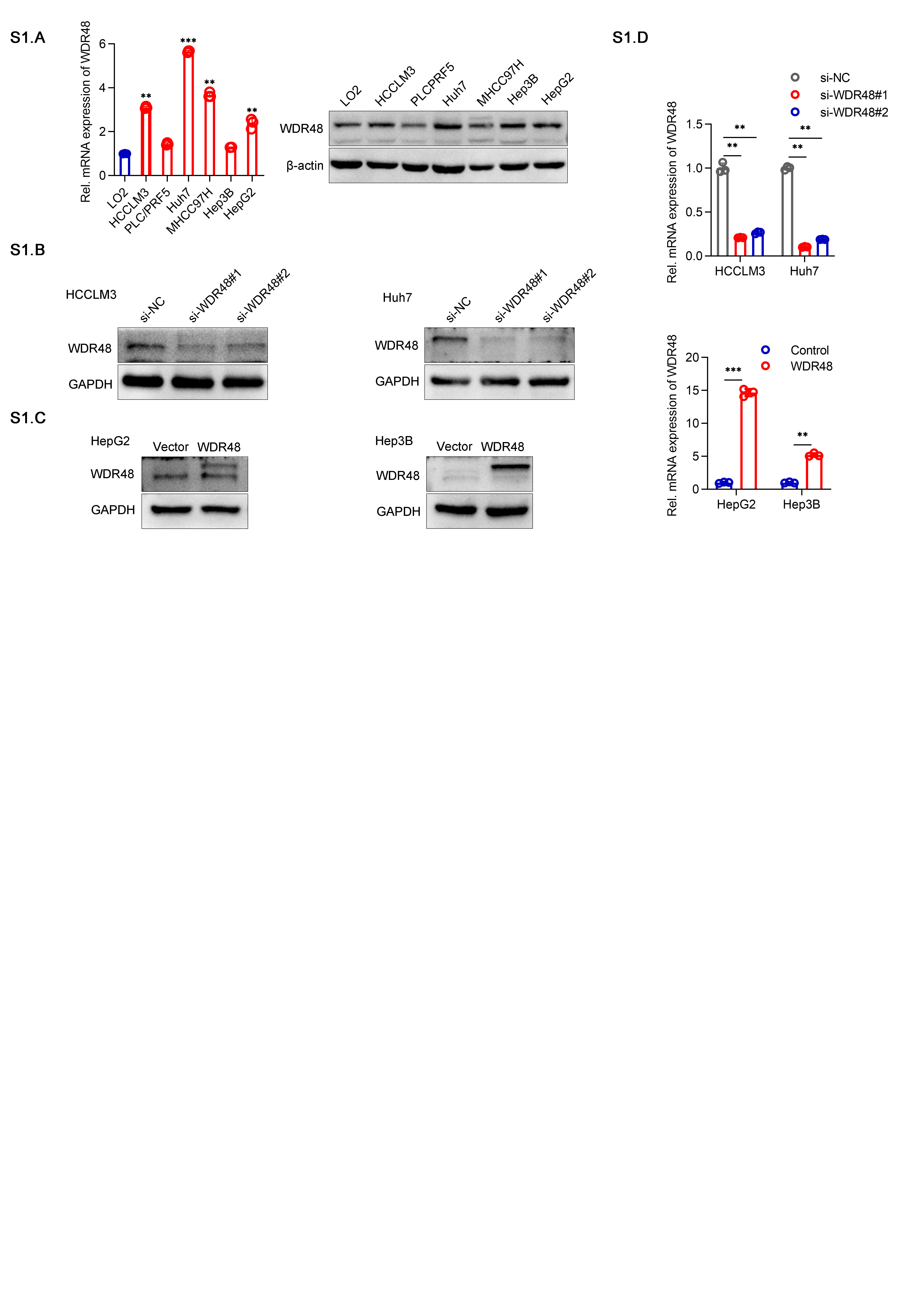

Supplement: Supplementary file 1 — Figure S1 [file JCMM-26-5755-s002.jpg]

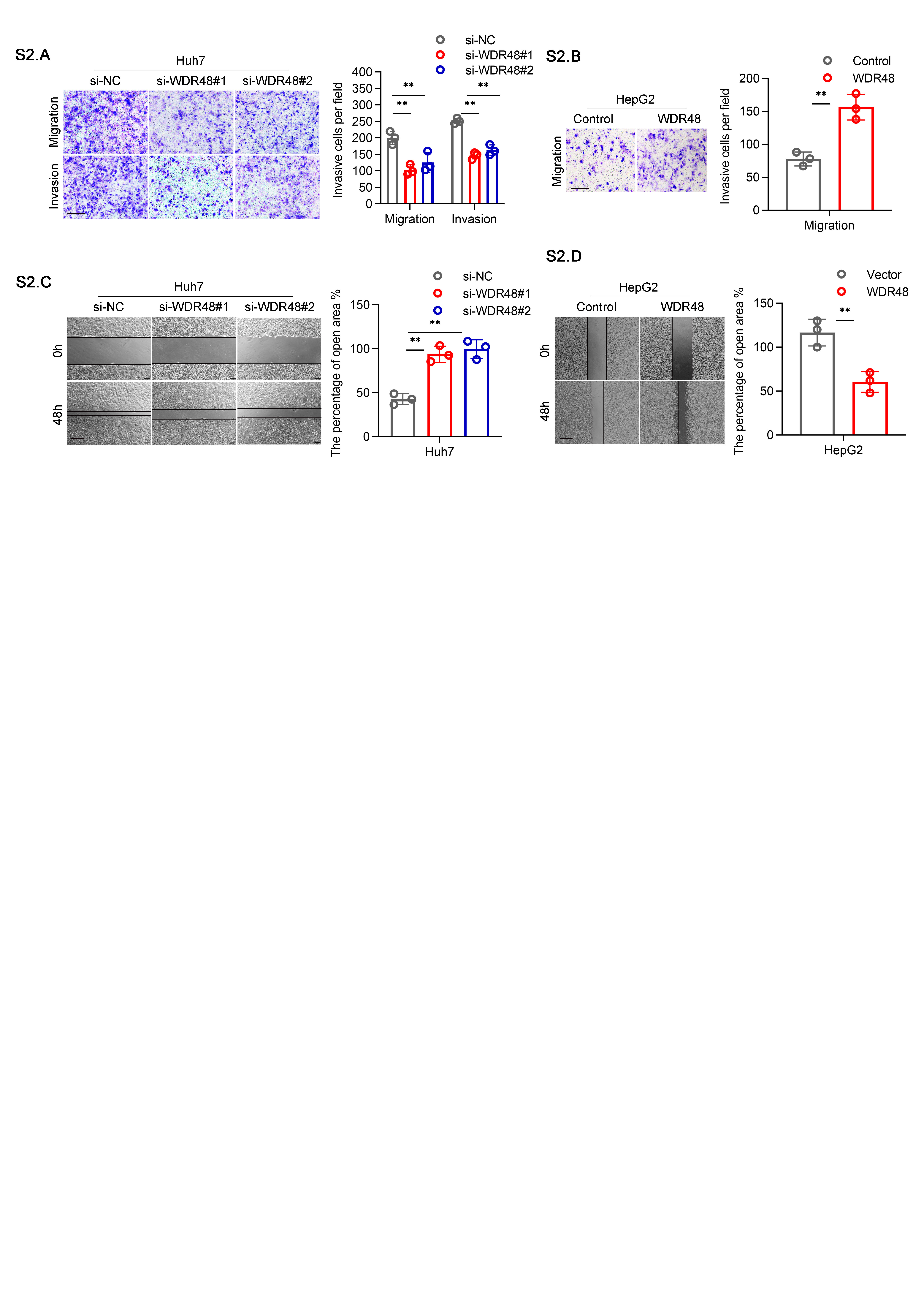

Supplement: Supplementary file 2 — Figure S2 [file JCMM-26-5755-s001.jpg]
