# Supplementary material for: WD repeat domain 48 promotes hepatocellular carcinoma progression by stabilizing c‐Myc
Source: J Cell Mol Med. 2022 Nov 20;26(23):5755–66. doi: 10.1111/jcmm.17583 (PMC9716212; doi:10.1111/jcmm.17583)
Supplement: Supplementary file 3 — Table S1‐S4 [file JCMM-26-5755-s003.docx]

**Supplementary Table S1.**

**Correlation of WDR48 expression with clinicopathological characteristics of patients with HCC.**

| **Characteristics** | **n** | **WDR48 expression** | | ***P*** |
| --- | --- | --- | --- | --- |
|  |  | **Low** | **High** |  |
| **Gender** |  |  |  |  |
| Male | 80 | 40(50.00%) | 40(50.00%) | 1.000 |
| Female | 10 | 5 (50.00%) | 5 (50.00%) |  |
| **Age** |  |  |  |  |
| <50 | 37 | 19(51.35%) | 18(48.65%) | 0.830 |
| ≥50 | 53 | 26(49.06%) | 27(50.94%) |  |
| **AJCC Stage** |  |  |  |  |
| I | 63 | 27(42.86%) | 36(57.14%) | **0.038** |
| II-III | 27 | 18(66.67%) | 9(33.33%) |  |
| **Tumor size(cm)** |  |  |  |  |
| <4.5 | 48 | 19(39.58%) | 29(60.42%) | **0.035** |
| ≥4.5 | 42 | 26(61.90%) | 16(38.10%) |  |
| **Vital Status** |  |  |  |  |
| Alive | 58 | 24(41.38%) | 34(58.62%) | **0.028** |
| Dead | 32 | 21(65.62%) | 11(34.38%) |  |
| **Recurrence** |  |  |  |  |
| No | 41 | 15(36.59%) | 26(63.41%) | **0.020** |
| Yes | 49 | 30(61.22%) | 19(38.78%) |  |
| **Cirrhosis** |  |  |  |  |
| Absent | 9 | 5 (55.56%) | 4 (44.44%) | 0.699 |
| Present | 80 | 39(48.75%) | 41(51.25%) |  |
| **HBsAg** |  |  |  |  |
| Absent | 19 | 10(52.63%) | 9 (47.37%) | 0.754 |
| Present | 70 | 34(48.57%) | 36(51.43%) |  |
| **ALT level(U/L)** |  |  |  |  |
| <50 | 60 | 29(48.33%) | 31(51.67%) | 0.764 |
| ≥50 | 29 | 15(51.72%) | 14(48.28%) |  |
| **AFP level(ng/L)** |  |  |  |  |
| <400 | 57 | 27(47.37%) | 30(52.63%) | 0.602 |
| ≥400 | 32 | 17(53.12%) | 15(46.88%) |  |
| **Histological Grade** |  |  |  |  |
| I-II | 66 | 29(43.94%) | 37(56.06%) | 0.057 |
| III | 24 | 16(66.67%) | 8 (33.33%) |  |

**Supplementary Table S2.**

| **The sequences used in this study.** | | |
| --- | --- | --- |
| Gene | No. | Target sequences |
| WDR48 | 1 | GGTCGAGACTCTATCATAA |
|  | 2 | GCAGAGATGTATAGCAACA |
|  | 3 | GTATCAGGGTCCACTGAAA |
| c-Myc | 1 | CAGAAATGTCCTGAGCAAT |
|  | 2 | GGTCAGAGTCTGGATCACC |
|  | 3 | GATGAGGAAGAAATCGATG |

**Supplementary Table S3.**

| **The primers used in this study.** | | |
| --- | --- | --- |
| Primers name |  | Sequence (5’-3’ ) |
| WDR48 | Forward | AGAAGTACAACCGAAATGGAGTC |
|  | Reverse | ACAATGTCGTTTACCCAATCAGT |
| c-Myc | Forward | GCATCGTTTTCCTCCTTATGCC |
|  | Reverse | CAAACCGCATCCTTGTCCTG |
| β-actin | Forward | ACAGAGCCTCGCCTTTGCC |
|  | Reverse | GATATCATCATCCATGGTGAGCTGG |

**Supplementary Table S4.**

| **A list of antibodies used for Western blot, IHC, IF, Co-IP.** | | | | |
| --- | --- | --- | --- | --- |
| Antibodies | Company | Cat.No | Mol weight (kDa) | Dilution |
| WDR48 | Signalway Antibody | 30739-2 | 76 | 1:1000(WB)  1:100(IHC) |
| c-Myc | Proteintech | 10828-1-AP | 50 | 1:1000(WB)  1:100(IF)  4.0µg(IP) |
| CCND1 | Proteintech | 60186-1-Ig | 36 | 1:5000(WB) |
| E-cadherin | Proteintech | 60335-1-Ig | 120 | 1:2000(WB) |
| N-cadherin | Proteintech | 66219-1-Ig | 130 | 1:2000(WB) |
| Vimentin | Proteintech | 10366-1-AP | 54 | 1:2000(WB) |
| FLAG | Sigma | F1804 | - | 1:100(IF) |
| GAPDH | Bioworld | pAb AP0063 | 36 | 1:5000(WB) |
